# Barriers and facilitators to lifestyle behavior change and goal setting in adolescents with polycystic ovary syndrome

**DOI:** 10.3389/fnut.2025.1628853

**Published:** 2025-11-24

**Authors:** Manasa Gadiraju, Joy Y. Kim, Erin M. Green, Alexandra MacMillan Uribe, Honghui Chang, Tania S. Burgert, Melissa D. Olfert, Heidi Vanden Brink

**Affiliations:** 1 Department of Surgery, University of Vermont Medical Center, Burlington, VT, United States; 2 Department of Nutrition, College Station, Texas A&M University, College Station, TX, United States; 3 Division of Nutritional Sciences, Cornell University, Ithaca, NY, United States; 4 Institute for Advancing Health Through Agriculture, Texas A&M AgriLife Research, Dallas, TX, United States; 5 Endocrinology, Children's Mercy Kansas City, Kansas City, MO, United States; 6 Davis College of Agriculture and Natural Resources, Program of Human Nutrition and Foods, School of Agriculture and Food Systems, West Virginia University, Morgantown, WV, United States

**Keywords:** registered dietitian nutritionist, RDN, PCOS, diet, qualitative research, goals, adolescence

## Abstract

**Purpose:**

To identify barriers and facilitators to lifestyle modifications and goal setting and characterize goal setting for adolescents with polycystic ovary syndrome (PCOS).

**Methods:**

Retrospective chart review of registered dietitian nutritionist (RDN) nutrition notes from 118 adolescents with PCOS 11–21 years evaluated at a multi-specialty PCOS clinic. Goals, barriers, and facilitators were coded using open coding and *a priori* objectives and characterized for emerging themes using qualitative content analysis.

**Results:**

Five major themes of barriers and facilitators to behavior change emerged: interest and motivation, family involvement, resources and food environment, taste preferences, and self-efficacy. RDNs set ≥3 goals with 52%, 2 goals with 35%, 1 goal with 9%, and no goals with 3% of adolescents with PCOS. Goals covered three major themes: incorporation of the United States Department of Agriculture (USDA) MyPlate model, modifying carbohydrate intake, and increasing physical activity.

**Conclusion:**

The facilitators and barriers identified through our analysis are both similar and different to those reported in adolescents with obesity and women with PCOS, likely due to differences in condition specific contexts and life stage. The goals recorded by RDNs reflect a desire to increase diet quality; however, too many goals may have been set on average. Overall, adolescents with PCOS report intrapersonal, interpersonal, and environmental barriers and facilitators that may affect their ability to establish and act upon goals.

## 1 Introduction

Polycystic ovary syndrome (PCOS) is a highly prevalent endocrine disorder affecting approximately 6%−10% of adolescents living in the United States (1). PCOS is associated with multisystem comorbidities including obesity, hypertension, cardiovascular disease, and infertility, and negative impacts on mental health and overall wellbeing (2–4). Although PCOS is most often diagnosed in adulthood (5), PCOS and its multisystem comorbidities manifest during adolescence (6). Therefore, diagnosis and intervention in adolescence are critical to mitigating the lifelong chronic disease burden of PCOS.

Lifestyle intervention is the first-line recommendation for management of PCOS (7). The 2023 International Evidence-based PCOS Guidelines recommend lifestyle modification, defined as exercise or diet with exercise or behavior modification, for all individuals with PCOS to improve metabolic health (7). While several dietary interventions have been shown to improve reproductive (8–10) and metabolic symptoms (10, 11) of PCOS, there is currently no ideal dietary pattern for PCOS and none specifically for adolescents with PCOS (7, 12).

Registered Dietitian Nutritionists (RDNs) can serve a central role in providing nutrition counseling and dietary support for PCOS. However, RDNs are not routinely involved in patient care for adults with PCOS (13–15), and less is known about the involvement of RDNs in adolescents diagnosed with PCOS (15). One approach RDNs use to support lifestyle behavior through the nutrition care process is goal setting (16). Goal setting theory is a proven and effective method in nutrition counseling for adults and adolescents that requires an understanding of barriers and facilitators toward behavior change to define achievable, realistic goals (17–20). For adults with prediabetes and type 2 diabetes, goal setting has been validated as an effective strategy for glycemic control and weight management and weight loss when coupled with patient participation and socially supportive environments (21, 22). Abstract reasoning emerges during adolescence, which makes goal setting an attractive tool to utilize during this stage of development (23). It is important to note that goals set by adolescents are unique to this lifespan period, and distinctive from those set by adults, warranting separate research in its effectiveness in this population (24). For example, goal-setting has been investigated with tools such as the EatFit intervention and appears to be an effective strategy for lifestyle modification and behavior change during adolescence (25). As such, goal setting may be a useful tool for nutrition counseling of adolescents with PCOS and remains an understudied application.

Dietary management and support for PCOS in adolescents is poorly understood. Adolescence represents a developmental transition toward food autonomy; however, this can be complicated by dependency on adults for food procurement and food choice (26). Consequently, adolescence as a window for lifestyle interventions is also likely to be distinct from adulthood (27). Considering that adolescents have the poorest diet quality among all other age groups in the United States (28) and maintain poor compliance lifestyle interventions (29), there is a need to understand how to effectively implement dietary interventions in this age group. To gain insight into the current state of nutritional support for adolescents with PCOS, we conducted a retrospective chart review of nutrition notes recorded by RDNs who counseled adolescents seen for PCOS at a multi-disciplinary PCOS clinic. Our objectives were to identify recorded barriers and facilitators to lifestyle behavior change in adolescents with PCOS and describe the number and nature of goals between the RDN and adolescent with PCOS.

## 2 Methods

### 2.1 Data collection

A retrospective chart review was conducted at a tertiary pediatric medical center in a Midwestern state in the United States and included adolescents evaluated for PCOS who concurrently met with an RDN at a multidisciplinary PCOS-adolescent medicine clinic (MAPP) between 2015 and 2020. Further detail on this participant sample has been previously described (30). Briefly, participants included in the chart review were female adolescents 11–21 years of age who met diagnostic criteria for PCOS based on the 2018 International Guidelines (12) and were consecutively seen by MAPP clinic providers. Irregular menstruation was self-reported via open-ended questions asked by adolescent medicine physicians as documented in the electronic medical record (EMR). Menstrual irregularity was defined as irregular menstruation at >1-year post-menarche or primary amenorrhea as recorded in the medical record. Clinical hyperandrogenism was defined as a Ferriman–Gallwey (FG) hirsutism score >4(12) assessed via physical examination by either an endocrinologist or adolescent medicine physicians and documented in the EMR. Biochemical hyperandrogenism was defined as a total testosterone ≥40 ng/dL or free testosterone >1.09 ng/dL. The classification of PCOS for the purpose of this study was made by the research team after data extraction from the medical record. Patients with previous diagnoses of PCOS by outside clinicians were included if diagnostic criteria were confirmed through medical records or repeat labs. Adolescents with PCOS taking oral contraceptive pills at the time of the MAPP visit were included if a PCOS diagnosis could be established prior to the MAPP clinic visit using referral history and previous laboratory values completed prior to initiation of the hormonal medications.

Nutrition notes recorded by one of eight RDNs who conducted nutrition visits with patients seen for PCOS evaluation were extracted and de-identified from the EMR. Due to the exploratory nature of the study, a sample size calculation was not conducted. In total, 118 nutrition notes representing 118 unique patients seen at the multi-disciplinary clinic were included in the final analysis. Of the 118 patients, 29/118 (25%) had seen an RDN previously in the hospital system. The majority of patients (103/118, 87%) were seen by three RDNs. The nutrition note from the first MAPP visit was included per patient in the analysis. Nutrition notes followed a standardized format utilized by the clinic, either using a weight management (*n* = 108) or outpatient nutrition plan of care note (*n* = 10) (Table 1). Study data were collected and managed using REDCap (Research Electronic Data Capture). REDCap is a secure, web-based software platform designed to support data capture for research studies (31, 32).

**Table 1:** Outline of a weight management nutrition note.

**Section**	**Description**
Subjective history	• Information on if patient attends school and who patient lives with • Pre-populated and free-text options to record subjective information on patient's health history, nutrition history, current health concerns, observed psychosocial health, etc.
Food/activity history	• Free-text typical day of eating for the patient, describing beverages, meals, and snacks • Amount and type of physical activity patient engages in
Objective history	• Anthropometry is auto populated from EMR • Pre-populated options to select and free text to include relevant medical information (e.g., medications or supplements, adverse reactions, lab comments, additional anthropometry, estimated energy recommendation, etc.) from EMR
Nutrition diagnostic details	• One or more nutrition diagnoses written using pre-populated options following a Problem, Etiology, and Signs/Symptoms (PES) format.
Treatment/intervention details	• One or more interventions implemented from pre-populated choices • Education materials provided to the patient from pre-populated choices • Evaluation of patient understanding using pre-populated options • Free-text intervention comments that expand upon intervention details
Food plan *(Optional)*	• Recommended daily nutrition plan for the patient
Goals	• Free-text list of goals set
Other	• Notes if family verbalized understanding and nutrition contact information was provided • Time spent with patient/family (including documentation time)

Weight management templates were the predominant nutrition note template used. However, the Outpatient Plan of Care was used in a subset of patients (n = 10).

### 2.2 Data analysis

Conventional and directed qualitative content analysis (33) using Zhang and Wildemuth's process (34) was applied to the nutrition notes. In the preparation phase, one investigator extracted and deidentified nutrition notes and uploaded notes into a qualitative and mixed methods coding software, Dedoose (version 9.0.107) (35) for analysis. During the organization phase, codes were first generated based on the *a priori* objectives of the authors, and two investigators reviewed approximately 30% of the recalls, open-coding based on emerging themes. Initial *a priori* objectives included understanding how barriers and facilitators to healthy behaviors are described to a RDN by patients and/or their caregivers, how RDNs construct goals with their patients during counseling interventions, how knowledge of PCOS influences patients, and the characteristics of adolescent patients' reported diets during RDN counseling sessions. Codes were then categorized and grouped to create an initial code book. After one investigator completed coding of all notes, four members of the research team reviewed the coding and field notes to update the codebook based on newly emerging themes, redundancy in codes, and more appropriate categories. All nutrition notes were reviewed again, and coding was updated to reflect the revised codebook by one investigator. Thereafter, a fifth investigator reviewed approximately 30% of the notes to reconcile remaining discrepancies and finalize the codebook. Once the codebook was finalized, the fifth investigator reviewed the remaining 70% of notes to ensure consistency in application of codes. After coding of all notes was completed, codes were examined to identify emerging themes related to goal setting and barriers and facilitators to lifestyle behavior change. We first identified themes that were more consistent and concentrated than others, which were then identified as stronger themes, and second identified those that emerged but with less consistency.

All study activities were approved by the Institutional Review Board of Midwestern Hospital. Due to the nature of the retrospective chart review, permission/assent or consent were not required.

## 3 Results

### 3.1 Characteristics of participants

A total of 118 nutrition notes from individual patients were coded and included in the analysis. The demographics of the participating adolescents are presented in Table 2. Twenty adolescents were taking oral contraceptives at the time of MAPP visit. One hundred and three adolescents were less than 18 years of age. A sub-group analysis of those participants < 18 years is included in Supplementary Table S1 and Supplementary Figures S1, S2; however, the analyses were similar to the full sample, therefore the results presented herein reflect the full sample.

**Table 2:** Demographic characteristics of adolescents with PCOS seen by an RDN at a multidisciplinary PCOS clinic (*n* = 118).

**Demographics**	**Mean (range)**
**Age (y)**	16.1 (11.2–21)
**Race and ethnicity**	***n*** **(%)**
Non-Hispanic White	52 (44.1%)
Hispanic White	29 (24.6%)
Black or African American	21 (17.8%)
Asian	4 (3.3%)
Multiracial or other	12 (10.2%)
**BMI category**	
5–84th %ile or 18.6–24.9 kg/m^2^	14 (11.9%)
85–89th %ile or 25–29.9 kg/m^2^	21 (17.8%)
95–99th %ile or >30 kg/m^2^	83 (70.3%)

### 3.2 Barriers and facilitators to lifestyle behavior change

**Figure 1:**
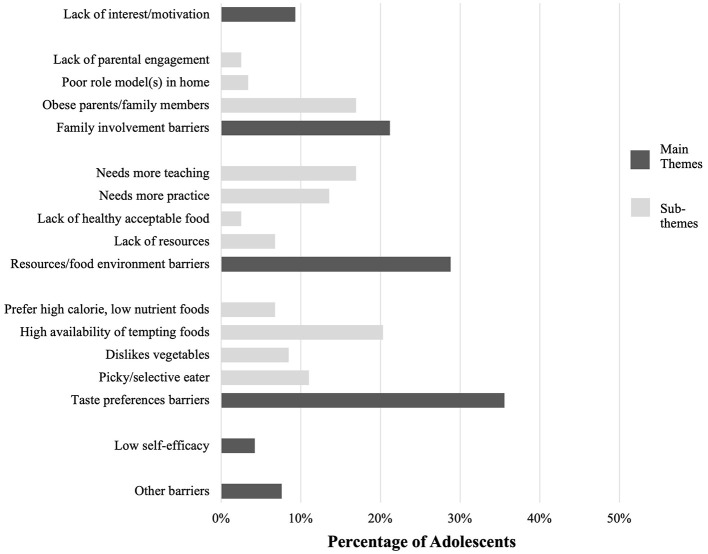
Perceived barriers to behavior change and goal setting in adolescents with PCOS during their initial visit with an RDN at the MAPP clinic.

**Figure 2:**
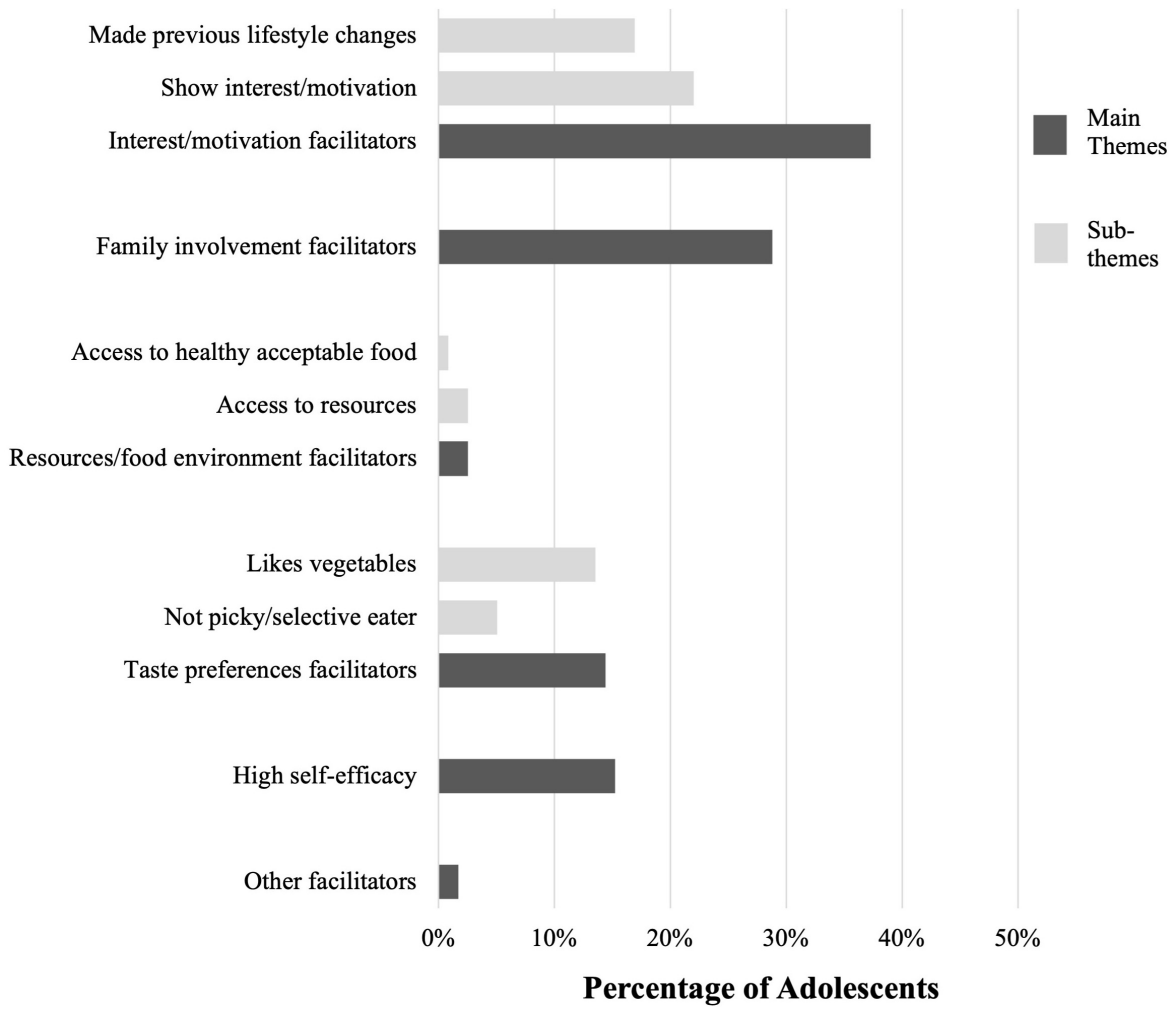
Perceived facilitators to behavior change and goal setting in adolescents with PCOS during their initial visit with an RDN at the MAPP clinic.

Five overarching themes emerged that summarized both barriers and facilitators: interest and motivation, family involvement, resources and the food environment, taste preferences, and self-efficacy (dark gray bars, Figures 1, 2). The relative distribution and sub-themes, or child codes, of each varied substantially (light gray bars, Figures 1, 2).

#### 3.2.1 Barriers

Barriers to lifestyle changes and/or perceived healthy living were captured via qualitative thematic analysis of the RDN notes, which are summarized in Figure 1. The most frequently cited barrier affecting 35.6% of adolescents related to taste preference barriers, including being picky eaters (11.0%), disliking vegetables (8.5%), or a high availability of tempting foods (20.3%). The next most common barrier, emerging in 34 patients (28.8%), was resource-related, including a lack of resources related to time restraints and a lack of healthy and enjoyable foods available to the patient at home and school. We also coded notations from RDNs who recorded adolescents and/or parents as requiring “more teaching” (16.9%) or “more practice” (13.6%) as resource-related, which were interpreted as a perceived lack of nutrition and/or health literacy. The third most common barrier, arising in 25 patients (21.2%), was related to family involvement. Sub-themes under family involvement included the RDN documenting parents or family members with obesity (16.9%), poor role models in the home (3.4%), and lack of parental engagement or a belief that lifestyle change would be helpful in managing PCOS symptoms (2.5%) which indicates there may be a lack of belief in the efficacy of further diet and lifestyle recommendations for PCOS among parents of adolescents with PCOS.

Five patients (4.2%) expressed they felt unsure about their ability to implement recommendations provided by the RDN, indicating low self-efficacy. Some sessions elicited emotional responses, such as feelings of being overwhelmed.

#### 3.2.2 Facilitators

The most frequently observed facilitator was patient-driven interest and motivation, seen in 44 patients (37.3%). Statements related to motivation were either made by patients or family and noted by the RDN or directly observed by the RDN (Figure 2). Statements reflected an interest in learning about diet quality and motivation to develop healthy habits (22.0%), as well as reported establishment of previous lifestyle changes and healthy habits (16.9%), such as working on incorporating more fruits and vegetables in the diet or limiting processed foods and incorporating regular exercise.

Parental involvement with positive lifestyle change was the second most frequently observed facilitator, documented in 34 patient notes (28.8%). Many adolescents who previously made changes to their eating and lifestyle habits stated that these changes were made together with their parent(s) and/or entire family. Parents also reported making changes to their own diet and lifestyle habits, independent from the health concerns of their child, which has influenced their motivation to implement positive changes for their child.

Taste positivity or being open to trying new foods (5.1%) or enjoying the taste of vegetables (13.6%), was recorded in the notes of 17 (14.4%) adolescents with PCOS. Overall, perceived behavioral competence which we categorized as self-efficacy was more commonly recorded as being present than absent; 18 patients (15.3%) expressed positive prospects to the goals they set with the RDN, indicating their confidence and willingness to apply suggested nutrition and physical activity changes.

### 3.3 Goal setting

Three or more lifestyle behavior goals were set between the RDN and adolescents with PCOS in the majority of patients during their first visit to MAPP (Table 3). Despite most visits being attended by one or both parents (105 visits), parental inclusion in the recorded goals was rare, observed only in 11 of the nutrition notes in this sample (Table 3). Of the goals set by the participants and RDNs, they aggregated around three themes: (1) MyPlate/plate model, (2) modifying carbohydrate intake, and (3) physical activity.

**Table 3:** Characteristics of goal setting and parental involvement at RDN visits.

**Visit Characteristics**	***n* (%)**
**Goals set per visit**	
0	4 (3.4%)
1	11 (9.3%)
2	41 (34.7%)
3+	62 (52.5%)
**Parent(s)/Legal Guardian(s) attended visit?**	
Yes	105/118 visits (89.0%)
No	13/118 visits (11.0%)
**Parent(s)/Legal Guardian(s) included in goal(s)?**	
Yes	11/118 visits (9.3%)
No	103/118 visits (87.3%)

#### 3.3.1 MyPlate/plate model

MyPlate, also generally known as the “plate model,” is a graphical depiction of a balanced meal based on the United States Department of Agriculture's Dietary Guidelines for Americans (36). RDNs recorded the incorporation of MyPlate in 63 (53%) of goals to guide healthy food choices and portion control, most often emphasizing non-starchy vegetables, whole grains, and lean protein.

#### 3.3.2 Modifying carbohydrate intake

Modifying carbohydrate intake was a common theme throughout the goals, which was included in 92 or 78% of all nutrition notes. Carbohydrate modification commonly focused on restricting simple carbohydrates, such as reducing intake of sugar-sweetened beverages and other sources of added sugars. Few RDNs recorded goals set with the adolescent which focused on adding complex carbohydrates (e.g., increase whole grains). Other recommended strategies given to adolescents with PCOS by the RDNs were to pair carbohydrates with other macronutrients (e.g., protein or fat) and reduce portion sizes of starchy carbohydrates at meals.

#### 3.3.3 Physical activity

Many nutrition notes included goals that targeted physical activity appearing in 87, or 74%, of all nutrition notes. Walking was often suggested as an introductory form of exercise. RDNs recorded setting goals that reflect going to the gym and participating in school sports, which appeared to be based more on adolescents' willingness and access to facilities and/or opportunity.

## 4 Discussion

Although lifestyle management is recommended as a first-line intervention strategy for women living with PCOS, how lifestyle changes are implemented with adolescents, who are at a dynamic and transitional life stage, are poorly understood. Our objective was to begin to understand and summarize current strategies implemented or proposed by RDNs in a clinical setting for adolescents with PCOS. We observed heterogeneity in the barriers and facilitators to lifestyle behavior change, with taste preferences representing the predominant barrier and motivation and interest in lifestyle behavior change representing the predominant facilitator. Most RDNs set 3 or more goals related to lifestyle behavior change during the session, and goal-setting did not vary significantly among different RDNs.

Facilitators for behavior change are similar to those reported in adolescents with obesity (37), but less similar to those reported by women with PCOS, possibly due to differences in life stage and a focus on specifically diet lifestyle changes vs. general PCOS management (38–40). The most prominently recorded facilitator in the present study related to the adolescent being interested in and motivated to make lifestyle changes, which is a shared facilitator seen in both adolescents with obesity (25) and women with PCOS (38, 39). Although it cannot be determined for participants that their expressed motivation was intrinsic versus extrinsic, goal commitment is influenced by the degree of intrinsic motivation (41). Therefore, participants who presented with intrinsic motivation may have a higher likelihood of attempting the goals set by them and the RDN and seeing more positive outcomes (41). The lack of similarities in other facilitators reported by adolescents in the present study may be due to the lack of studies assessing facilitators to dietary and lifestyle changes specifically in the context of PCOS management, vs. more general PCOS management (42–49), or possibly due to differences in life stages.

The barriers which emerged in our analysis align with other research in adults with PCOS (38, 39) and adolescents with obesity (37); the most common were related to taste preferences and a dislike for more nutrient-dense foods. Lim et al. described emotional and logistical barriers to behavior change, such as defeating thoughts and time, respectively (50). In our sample, financial resources and availability of healthy foods represent logistical barriers in our whereas taste preferences and self-efficacy may be considered emotional barriers. Logistical and financial barriers are important considerations in the evaluation of multidisciplinary PCOS clinics with RD counseling – this comprehensive care setting is multi-specialty and resource-heavy and requires favorable environmental and socioeconomic disposition of the adolescent for their participation. Taste preferences, and availability of nutrient-dense options for adolescents were prevalent barriers, reported in 38% of all participants, which may provide insight when sustainability of lifestyle change and compliance when conducting dietary interventions in adolescents. Lifestyle interventions among adolescents with PCOS (29), overweight or obesity (51–55), or type 2 diabetes (51–56) often report low compliance. When we consider the implications of our findings and others (39, 57) as they relate to lifestyle interventions, taste preferences and availability of nutrient-dense options for adolescents may contribute to poor compliance for adolescent lifestyle interventions (29). Few adolescent lifestyle interventions in PCOS directly address the diverse array of challenges that adolescents face; high compliance to lifestyle behavior change has been reported in studies that directly addressed logistical (e.g., financial, access to healthy options) barriers (58, 59).

The goals recorded by RDNs, broadly speaking, reflect a desire to increase diet quality, which provides two insights. First, the shift to increase diet quality suggests that diet quality was suboptimal prior to the RDN encounter and PCOS diagnosis. While analysis of dietary intake is outside the scope of this dataset, efforts to increase diet quality are consistent with a recent systematic review and meta-analysis, which reported lower diet quality in women with PCOS (60). Since adolescents have poorer diet quality compared to other age groups (28), we cannot discount that efforts to increase diet quality were attributed to a generally low diet quality, rather than PCOS *per se*. Second, while we cannot overlook the numerous barriers, most participants exhibit some evidence of readiness to change, either in expressed or observed interest or motivation and changes already made in the home. We did not assess adherence to SMART goals in our analysis of the goals, however we noted that most goals were specific and measurable (e.g., “make $\frac{1}{2}$ the plate vegetables at dinner” rather than “eat more vegetables”), however few were explicitly time-bound, thereby not fully aligning with the SMART framework (61).

Our finding that most RDNs set three or more goals with adolescents was unexpected. We cannot infer the rationale behind setting 3 or more goals; however, it is plausible that high goal setting with the RDN reflects intention for planned behavior change (62, 63). While strategies for effective goal setting for PCOS are poorly understood, the success of goal setting for lifestyle interventions has been studied in other populations. In a study contrasting adolescent-parent dyads who completed a lifestyle intervention for overweight and obesity versus the non-completers, reported that too many goals was identified as barrier for 50% of the adolescent non-completers surveyed (64). For adults with type 2 diabetes, setting no more than three goals that are strongly aligned with patients' preferences led to greater weight loss and goal attainment (22). With that in mind, three goals set during the initial diet counseling visit may be too many considering previous literature and adolescent life stage and suggest that future research is needed to clarify optimal goal-setting strategies in the management of PCOS in adolescents.

Despite the high prevalence of adolescents with overweight or obese body habitus in the participant sample, there was little emphasis on weight reduction in the goals recorded by the RDNs. This approach may have been the result of acknowledgment of weight stigma that is commonly reported in individuals with PCOS (65). The 2023 International Evidence-based PCOS Guidelines acknowledge the negative biopsychosocial impacts of weight stigma in healthcare that many women with PCOS experience and recommends that healthcare professionals be aware of weight bias in their clinical practice, and suggest weight-inclusive care (7). Although the guidelines acknowledge limited research on weight stigma in adolescents with PCOS (7) experiences with weight stigma are prevalent in adolescents with obesity (66). Intervention messaging focused on a healthy lifestyle has been seen to be more comprehensive and considerate of both physical and mental components of health in adolescents (67) with weight-neutral interventions presenting few differences in weight-related outcomes in adolescents and young adults when compared to conventional weight-focused interventions. This may be partially because promotion of healthy diet and physical activity is likely to lead to weight reduction, although more research is required to determine long-term efficacy (68), particularly in adolescents with PCOS.

Parent involvement emerged as both a barrier and facilitator in the nutrition notes, yet we noted that parents were infrequently included in the recorded goal-setting strategies, which was unexpected. We are unable to infer as to why parents were or were not included in goals, however it is plausible that there was a deliberate decision to prioritize the adolescent patients' autonomy. We were also unable to identify whether the parent or adolescent noted the relevant family involvement barriers or facilitators. However, our observations are consistent with another study, in that parental engagement was both a significant barrier and facilitator in adolescents with obesity (37), underscoring the complex and unique parental responsibility in teenagers adopting healthy habits and lifestyle changes (69). Degree of parental engagement may complex and more personalized; in one study teenagers' disfavor of high parental monitoring and conflicts between parent and adolescent contributed to non-compliance of a lifestyle obesity management intervention (64). However, others report that parental involvement could help to support adolescents' autonomy changes while positively influencing their establishment of a balanced lifestyle, decision-making, and healthy identity (69). Future studies should further explore questions on the effective ways to support parents in their role during their child's adolescent developmental period.

This study is strengthened by its inclusion of diverse populations and addresses a paucity of data in characterizing goal-setting and related factors that facilitate or discourage behavior change in adolescents with PCOS. The analysis of nutrition notes is a unique approach, providing insight into the RDN's role in medical management of PCOS. Although the content of nutrition notes may be inherently limited due to structure guidelines, analyzing nutrition notes via content analysis provides an essential framework to establish subsequent research questions. The diverse cohort of adolescents in this study yields multiple distinct perspectives of goal setting, which is of importance considering the racial and ethnic health disparities that affect women living with PCOS (70). However, we acknowledge the study was conducted in a single Midwestern clinic, which may limit the generalizability of the findings as regional environmental influences such as the local food environment and culture may reduce the applicability of this sample the population broadly. Other limitations of this study includes the cross-sectional design and retrospective and subjective nature of nutrition notes which are prone to implicit biases of the documenting RDNs and can only capture what the RDN has selected to notate and may not include other relevant details during visits. Many nutrition notes are also standardized to an extent as they utilize healthy weight or overweight/obesity nutrition note templates, rather than fully free-form or PCOS-specific templates, which may limit the amount of detail or uniqueness across patients provided.

In summary, adolescents with PCOS report several intrapersonal, interpersonal, and environmental barriers and facilitators that may affect their ability to establish and enact these goals. Additional prospective research is needed to establish optimal goal-setting in adolescents with PCOS, as too many goals may discourage positive action. Future research should explore the impact of goal setting strategies and nutritional counseling on long-term behavior change and PCOS outcomes in adolescents with PCOS.

## Author's note

Portions of this research were presented as abstracts at the Society for Nutrition, Education and Behavior Annual Meeting, the ISBNPA Annual Meeting, and at Children's Mercy Hospital Kansas City.

## Data Availability

The datasets presented in this article are not readily available however the data that support the findings of this study may be made available from the corresponding author upon reasonable request and appropriate institutional agreements. Requests to access the datasets should be directed to heidi.vandenbrink@ag.tamu.edu.
