# Supplementary material for: Barriers and facilitators to lifestyle behavior change and goal setting in adolescents with polycystic ovary syndrome
Source: Front Nutr. 2025 Nov 24;12:1628853. doi: 10.3389/fnut.2025.1628853 (PMC12684102; doi:10.3389/fnut.2025.1628853)
Supplement: Supplementary file 1 [file Table_1.docx]

**Supplementary Materials**

**Table S1. Characteristics of goal setting and parental involvement at RD visits for participants <18 years of age**

| **Visit Characteristics** | **n (%)** |
| --- | --- |
| **Goals set per visit** |  |
| 0 | 3 (2.9%) |
| 1 | 10 (9.7%) |
| 2 | 35 (34.0%) |
| 3+ | 55 (53.4%) |
| **Parents attended visit?** |  |
| Yes | 91/103 visits (88.3%) |
| No | 12/103 visits (11.7%) |
| **Parents included in goal(s)?** |  |
| Yes | 12/103 visits (11.7%) |
| No | 89/103 visits (86.4%) |

**Figure S1. Perceived barriers to goal setting in adolescents <18 years with PCOS during initial RDN visit**

**Figure S2. Perceived facilitators to goal setting in adolescents <18 years with PCOS during initial RDN visit**
